# Effectiveness of Mobile-Based Learning for Nasogastric Tube Intubation Among Medical Students: A Randomized Controlled Trial

**DOI:** 10.3390/healthcare13050546

**Published:** 2025-03-03

**Authors:** Ming-Hsuan Wu, Chen-Ju Chen, Huan-Fang Lee

**Affiliations:** 1 Department of Internal Medicine, National Cheng Kung University Hospital, College of Medicine, National Cheng Kung University, Tainan 704302, Taiwan; 2 Department of Nursing, National Cheng Kung University Hospital, College of Medicine, National Cheng Kung University, Tainan 704302, Taiwan; 3 Department of Nursing, College of Medicine, National Cheng Kung University, Tainan 704401, Taiwan

**Keywords:** intubation, gastrointestinal, mobile applications, students, medical, education, undergraduate, clinical competence

## Abstract

**Background**: Nasogastric tube (NGT) intubation is a critical skill, but it comes with the blind nature of the procedure and its high failure rates. Resources restrict access to traditional training methods, such as simulations based on manikins. We developed a mobile-based application, the Mobile-based Hands-on Learning System for Nasogastric Tube Intubation (MoHoNGT), to enhance undergraduate medical students’ training in this essential procedure. **Methods**: This open-label, randomized controlled trial was conducted in a medical center between August and October 2020, with medical students expected to enter their clerkships. The MoHoNGT and control group were exposed to the traditional training course and a self-learning period. The MoHoNGT group received additional access to MoHoNGT. Training effectiveness was evaluated by measuring knowledge, self-confidence, and performance on an objective structured clinical examination (OSCE). Statistical analyses included descriptive statistics, chi-square tests, and *t*-tests. **Results**: Seventy-three medical students were recruited. Thirty-two were allocated to the MoHoNGT group. No between-group differences were observed regarding demographic data. Post-intervention results indicated that the MoHoNGT group revealed more pronounced improvements in both NGT intubation knowledge (38.75 vs. 21.46, *p* < 0.001) and the confidence scale (8.50 vs. 5.17, *p* = 0.04). Post-study scores for NGT intubation knowledge were also higher in the MoHoNGT group (69.06 vs. 49.02, *p* < 0.001). Additionally, participants in the MoHoNGT group demonstrated superior performance on the OSCE (98.81 vs. 91.18, *p* = 0.003). **Conclusions**: Employing MoHoNGT with traditional training methods significantly enhanced the knowledge, self-confidence, and skills in NGT intubation among undergraduate medical students. This approach addresses various limitations of conventional techniques, suggesting that mobile-based learning could be a potential strategy for medical education.

## 1. Introduction

Many hospitalized patients require nasogastric tube (NGT) intubation. Nonetheless, it is a blind technique, as the NGT tip cannot be visualized during the insertion procedure. The failure rate is reported to be approximately 50% for the first attempt [1,2]. Furthermore, iatrogenic complications are not infrequent, including tubing kinks, mucosal tears, bleeding, tracheal intubation, aspiration pneumonia, and death [3,4]. In order to prevent these adverse outcomes and enhance the intubation success rate, proper NGT intubation skills cannot be overemphasized [5].

Research has shown that simulation-based training with real-time feedback is an appropriate method to support motor skill acquisition, including NGT intubation [6,7]. Traditionally, physical manikins are employed. They provide medical students with opportunities for repeated practice, and protect patients from being harmed by inexperienced trainees. However, costs and storage space limit the number of manikins available, resulting in limits to the time each student can spend on a manikin. The opening hours of clinical skill centers and human resources, for expert supervision, also pose further limitations on practice opportunities for students. What is worse, fewer practice opportunities translates to a longer time gap between training and performing real-world procedures. It is well known that without frequent practice, clinical skills decay over time [8]. 

Mobile-based learning is a transformative education type that caters to the needs of both educators and learners. In today’s world, where smartphones have become nearly ubiquitous, mobile-based learning is both accessible and cost-effective, enabling learning anytime and anywhere [9,10,11]. Multi-touch displays and various built-in sensors support learners by simulating actual hands-on operations, making smartphones ideal tools for procedural learning. In addition, smartphones enable users to receive real-time feedback without costly and time-consuming in-person teaching. On the other hand, educators can analyze data collected by the software to strengthen each learner’s weak points and meet individual needs [12,13]. Finally, along with the advancement of mobile networks, all the above functions can be implemented into a cross-platform web application running directly in a web browser without specialized software.

Education in various fields has shown that mobile-based learning enhances teaching effectiveness, ranging from basic suturing techniques for nurse practitioners to nursing skills for nursing students, episiotomy skills for midwifery students, and interprofessional knowledge construction between medical and pharmacy students [12,14,15,16,17,18,19,20]. However, despite the existence of online modules and instructional videos on NGT intubation, to our knowledge, no studies employing mobile-based applications for undergraduate medical students’ NGT intubation skills training have yet been published. We hope to extend mobile-based learning to this field, thus bridging the gap in medical education. In this study, we unified a multidisciplinary group that included gastroenterologists, otolaryngologists, nurse practitioners, and information and learning technology experts. We developed a mobile-based web application, the Mobile-based Hands-on Learning System for Nasogastric Tube Intubation (MoHoNGT), to facilitate NGT intubation training for undergraduate medical students. The present study thus aimed to evaluate the effectiveness of MoHoNGT compared to traditional teaching methods through a randomized clinical trial.

## 2. Materials and Methods

### 2.1. Study Design and Participants

This study was a prospective open-label randomized controlled trial, taking place in a medical center in southern Taiwan from August to October 2020. We utilized random number generators in IBM SPSS Statistics 22.0.0 to assign participants to either intervention or control groups. With a desired power of 0.9 and a significance level of 0.05 for a two-tailed test, it was estimated that 31 participants would be required for each group. The effect size, 0.85, was determined based on a meta-analysis study focused on the effect of mobile learning [21]. Taking an attrition rate of 10% into account, the final sample size required for our study was 68.

The inclusion criteria were as follows: (a) being an adult aged 20 years or older, (b) medical students from a single university who were expected to enter their first-year clerkship in the next semester beginning in September 2020, and (c) owning and being able to use a smartphone. Those who (a) took a long leave that lasted for over a month in the current semester, (b) had significant visual or auditory impairments, (c) had previous experience in NGT intubation, and (d) declined to participate in the study would be excluded. All methods were carried out in accordance with the ethical standards of the institution and the Declaration of Helsinki. Informed consent for participation was obtained from all subjects involved in the study. The protocol was approved by the medical center’s Institutional Review Board (B-ER-109-194) on 17 August 2020. The study was also retrospectively registered on ClinicalTrials.gov (NCT05742659) on 21 December 2022. The retrospective registration was due to an inadvertent administrative oversight influenced by the COVID-19 pandemic in Taiwan, which led to various urgent practical concerns and a major shift in the clinical research personnel during the initial study period. However, all study procedures and outcomes were predefined, remained consistent with the original protocol, and should not reflect negatively on study integrity.

### 2.2. Study Procedures

**Figure 1:**
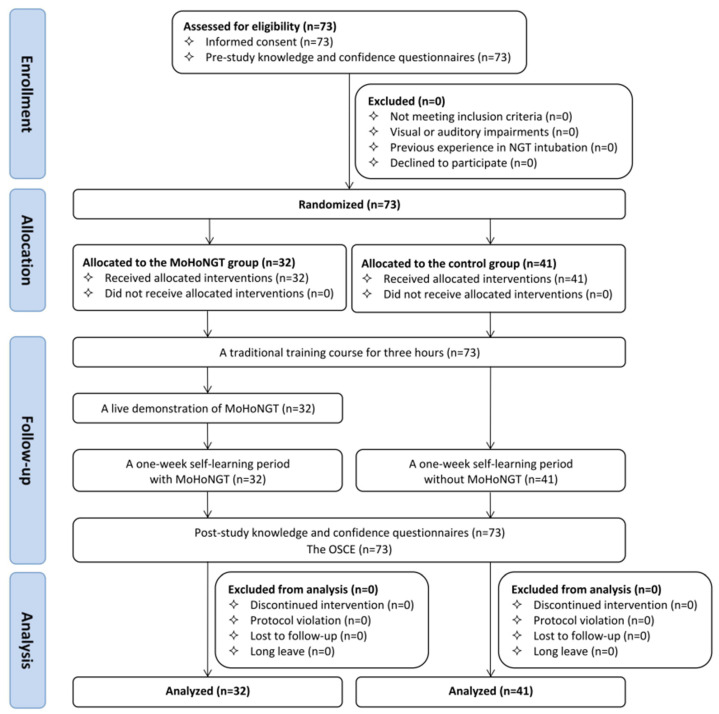
Consolidated Standards of Reporting Trials (CONSORT) flow diagram of participants. The flow diagram illustrates the study procedure. NGT: nasogastric tube; MoHoNGT: Mobile-based Hands-on Learning System for Nasogastric Tube Intubation; OSCE: objective structured clinical examination.

Figure 1 illustrates the entire procedure. At the beginning of the study, a researcher explained the recruitment details to the medical students and obtained their informed consent. The students then completed learning questionnaires (pre-study) to assess their eligibility, knowledge, and self-confidence in NGT intubation. Subsequently, eligible participants would be randomly divided into the intervention group (MoHoNGT group) and the control group. Both groups were exposed to the same traditional training course for three hours, which comprised lecture presentations, video demonstrations, real-world experience sharing, and hands-on practice using manikins. Afterward, the researcher presented a live demonstration of MoHoNGT to the participants in the MoHoNGT group, which lasted an hour. In addition, an online instructional video was also provided, which could be consulted anytime during the one-week self-learning period. The login usernames and passwords were then distributed to participants. In order to ensure controlled access to MoHoNGT, each participant was assigned a unique username and password combination. This not only allowed us to track their usage, but prevented unauthorized access by participants in the control group. After a one-week self-learning period, we terminated access to MoHoNGT from the server side. Both groups were then required to complete the learning questionnaires (post-study). Finally, all medical students took the objective structured clinical examination (OSCE), regardless of their eligibility for this study. The location where the OSCE was held adheres to the standard of Senior Professional and Technical Examinations for Medical Doctors, which serve as the national qualification exams for the Taiwanese medical license. The evaluators were attending physicians in the medical center who previously participated in the Senior Professional and Technical Examination for Medical Doctors as evaluators. They remained blinded to the study group assignments. All evaluators were trained and familiar with the standardized assessment framework. On the same day before the OSCE, all evaluators came together to reach a consensus on each marking scheme item. Medical students were assessed individually in ascending order according to their student identification numbers, irrespective of the study group assignments. Each of them was evaluated by one evaluator in each OSCE station.

### 2.3. Mobile-Based Hands-On Learning System for Nasogastric Tube Intubation (MoHoNGT)

**Figure 2:**
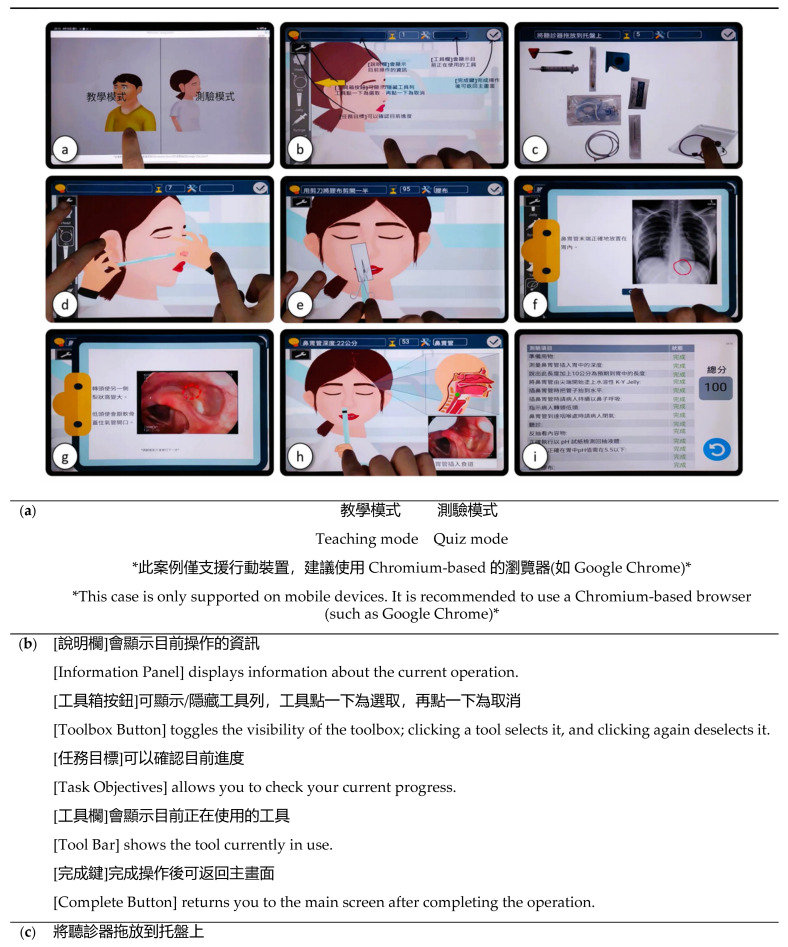

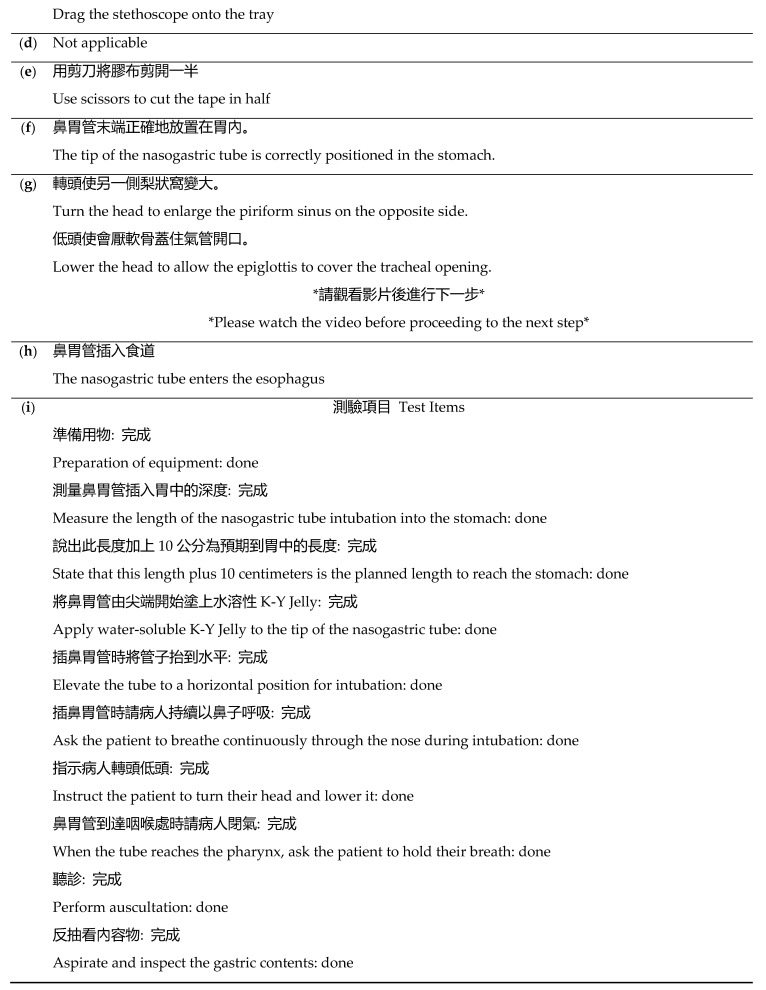

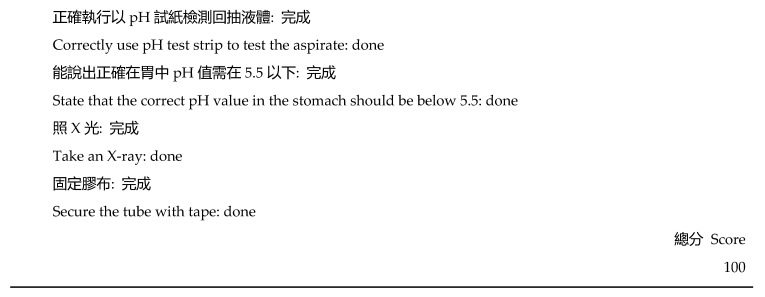
Screenshots showcasing key features of the Mobile-based Hands-on Learning System for Nasogastric Tube Intubation (MoHoNGT). The original figure is in Traditional Chinese, with its text translated into English as appended below. (**a**) The mode selection screen, with a learning mode (left) and a quiz mode (right); (**b**) an overview of elements and their corresponding functions on the main interface, including the central area, the toolbar (left), the timer (top), the button to view learning objectives (top left), etc.; (**c**) dragging the virtual stethoscope, which is required for NGT intubation, and dropping it onto a virtual plate; (**d**) stretching out on the virtual NGT to measure tubing from the bridge of the nose to the earlobe; (**e**) pinching in to manipulate the virtual scissors; (**f**) a step-by-step instruction screen displaying an X-ray image; (**g**) a step-by-step instruction screen playing an endoscopic video demonstrating the anatomy alterations of the pharynx when the volunteer turned and lowered their head; (**h**) the virtual–real fusion view, featuring a user passing the virtual NGT into the patient (left), a green dot denoting the position of the NGT tip on an illustration of sagittal anatomical structures (upper right), and video frames being playing in real time in conjunction with the corresponding location of the NGT tip (lower right); (**i**) The final score and the step-by-step diagnostic report that will show up after a user finishes the quiz mode.

To overcome the limitations of traditional NGT intubation training, our multidisciplinary team embraced mobile-based learning and developed MoHoNGT. It was built on the Deeply Interactive Virtual Environment, an application development platform utilizing an object-oriented visual programming language [22]. A member of our multidisciplinary team developed this proprietary, in-house platform. Its purpose is to streamline the creation of web-based, two-dimensional interactive content, as its low-code nature excludes the need for highly skilled or experienced developers. The integrated development environment and all applications built with this platform are hosted entirely online. Therefore, MoHoNGT is a cross-platform web application; it is readily accessible online via a web browser without the need to install third-party software. Figure 2 showcases key screenshots of MoHoNGT. Supplementary Material is a complete video walkthrough of MoHoNGT.

The mode selection screen is the initial interface that a user encounters upon logging in (Figure 2a). MoHoNGT comprises two distinct modes: the learning mode and the quiz mode. Rather than only providing static digital materials such as texts or videos, both modes are designed to represent the entire NGT intubation process from start to finish, including various hands-on operations in between. These range from preparing the necessary equipment (Figure 2c) and measuring the required length for intubation (Figure 2d) to performing the intubation itself (Figure 2h), confirming placement using various methods (Figure 2f), and securing the NGT with tape (Figure 2e). Throughout the learning process, users must actively interact with the application with multi-touch at each step, such as dragging equipment onto a virtual plate (Figure 2c), selecting the appropriate tool from the toolbar (Figure 2b), or stretching out on the virtual NGT to measure tubing from the bridge of the nose to the earlobe (Figure 2d). In the learning mode, step-by-step instructions (Figure 2f,g) are mandatory, assisting medical students in familiarizing themselves with both standard NGT intubation steps and the application’s user interface. The learning materials generally correspond to the context covered in our traditional training courses. In addition, we incorporated the results from our previous study into MoHoNGT [23]. In addition to virtual scenes and figures, we also loaded lots of real-life images, video footage, and audio, such as the appropriate appearance of stomach aspirate, an X-ray image of correct NGT placement (Figure 2f), and the “whoosh” sound when air is being injected into the stomach. One of the notable features is the virtual–real fusion view when a user passes the virtual NGT into the patient (Figure 2h). From a volunteer in our team, we documented full fiberscopy and esophagogastroduodenoscopy video recordings (Figure 2g). As the virtual NGT advances through the nasal cavity to the stomach, those video frames will be played in real time in conjunction with the corresponding location of the NGT tip. At the same time, a green dot moves along the route of NGT insertion on an illustration of sagittal anatomical structures, indicating the position of the NGT tip. We believe these designs would help medical students build a vivid and three-dimensional spatial concept, facilitating cognitive phase learning of this blind technique [24]. 

The quiz mode does not consist of questions in the traditional sense. Instead, it is a simulation of the entire NGT intubation scenario. The way in which the user interacts with the quiz mode is similar to that of the learning mode, with a few exceptions. First, the detailed instructions for NGT intubation are entirely absent. Additionally, the timing of each step is no longer restricted. Medical students are allowed to freely utilize any virtual tool at any point, giving them opportunities to make mistakes. Finally, after they finish in the quiz mode, a final score with an individualized step-by-step diagnostic report will be displayed (Figure 2i). Research has confirmed that immediate feedback effectively guides learners in recognizing mistakes and optimizing their performance over time [10,12,17,18].

### 2.4. Data Collection and Measurements

In addition to demographic information such as gender, smartphone usage, and previous e-learning experience in NGT intubation, some instruments were utilized to measure knowledge and skill capacity.

#### 2.4.1. The 10-Item Evaluation Questionnaire on NGT Intubation Knowledge

In order to ensure effective training outcomes, it is essential to evaluate participants’ cognitive understanding of NGT intubation, which aligns with the cognitive domain in Bloom’s taxonomy of learning objectives [25]. Furthermore, assessing knowledge represents level 2 (learning) of Kirkpatrick’s four-level framework for evaluating educational interventions [26]. Knowledge is foundational because NGT intubation carries risks of iatrogenic injury if performed without anatomical and procedural knowledge. Therefore, we developed a questionnaire comprising ten questions to assess knowledge of NGT intubation through a comprehensive literature review and clinical experience [23]. Three independent experts inspected the content to ensure its validity. The resulting questionnaire demonstrated a content validity index of 0.8. 

#### 2.4.2. The Confidence Scale

Confidence is critical in translating knowledge into practical skills and is essential for effective clinical performance. Being an affective factor in Bloom’s taxonomy, confidence directly influences learners’ willingness to perform a procedure, engage in clinical decision-making, and persevere through challenges [25]. By measuring confidence levels before and after training, we can determine whether the intervention effectively enhances self-efficacy among participants. Therefore, the study utilized the five-item confidence scale developed by Grundy to measure participants’ self-confidence in NGT intubation [27]. The confidence scale employs a five-point rating system, ranging from high uncertainty to complete certainty. In Grundy’s original study, the scale demonstrated a Cronbach’s alpha of 0.85. In previous studies, Cronbach’s alpha was reported as 0.973 and 0.90, respectively [17,28]. In the present study, Cronbach’s alpha was 0.96. 

#### 2.4.3. The Objective Structured Clinical Examination (OSCE)

Skill performance is the ultimate goal of clinical training. Assessing psychomotor skills through OSCEs is crucial for evaluating participants’ ability to perform NGT intubation competently in standardized settings. OSCEs are validated tools in medical education for objectively measuring clinical skills, aligning with Kirkpatrick’s level 3 (behavior) and the psychomotor domain of Bloom’s taxonomy [25,26]. This assessment also incorporates elements of preparation and verification that are important for patient safety and procedural success. Therefore, all medical students underwent the OSCE after the training courses. Those not eligible for this study could still attend the exam, but their data was excluded from the analysis. We developed the 16-item marking scheme based on the training courses, the literature review, and clinical experience [23]. Three independent experts evaluated the marking scheme and demonstrated a content validity index of 1.0. The score ranges from 0 to 100, with a higher score indicating greater proficiency in NGT intubation.

### 2.5. Statistical Analyses

We performed the analyses on the per-protocol population. The statistical analyses include descriptive statistics and chi-square tests for the demographic data, an independent-sample *t*-test to compare the differences between two groups, and a paired-sample *t*-test to assess within-group differences between pre-study and post-study scores. The software we adopted was IBM SPSS Statistics 22.0.0.

## 3. Results

### 3.1. Participants

A total of 73 medical students were enrolled in this study. Thirty-two of them were allocated to the MoHoNGT group, and the others to the control group. None of the participants were lost to follow-up, nor did they experience any protocol violations. The demographic data are presented in Table 1. Approximately two-thirds of the participants (65.8%) were male, with the majority (65.8%) using smartphones for over three hours per day. Most participants (87.7%) reported no previous e-learning experience in NGT intubation. No statistically significant differences were observed between the two groups, indicating that the participants were homogeneous in terms of demographic variables.

### 3.2. Capability for NGT Intubation

Table 2 compares pre-study and post-study outcomes regarding knowledge, self-confidence, and clinical skills. Regarding within-group differences, the MoHoNGT group demonstrated significant differences between pre-study and post-study scores in both NGT intubation knowledge (30.31 vs. 69.06, t = 13.04, *p* < 0.001) and the confidence scale (9.44 vs. 17.94, t = 6.39, *p* < 0.001). The results were similar to the control group, which also revealed significant within-group differences in the NGT intubation knowledge (11.49 vs. 16.66, t = 5.76, *p* < 0.001) and the confidence scale (11.49 vs. 16.66, t = 5.76, *p* < 0.001).

In terms of between-group differences, prior to the interventions, scores for the NGT intubation knowledge (30.31 vs. 27.56, *p* = 0.31) and the confidence scale (9.44 vs. 11.49, *p* = 0.15) did not differ significantly between the MoHoNGT group and the control group. However, post-study scores for NGT intubation knowledge were notably higher in the MoHoNGT group compared to the control group (69.06 vs. 49.02, *p* < 0.001). The MoHoNGT group also showed higher post-study scores on the confidence scale (17.94 vs. 16.66, *p* = 0.65), but this result was not statistically significant. Finally, the MoHoNGT group demonstrated more pronounced improvements in both NGT intubation knowledge (38.75 vs. 21.46, *p* < 0.001) and the confidence scale (8.50 vs. 5.17, *p* = 0.04).

Regarding the OSCE, the MoHoNGT group demonstrated superior performance to the control group, with a statistically significant difference (98.81 vs. 91.18, *p* = 0.003).

## 4. Discussion

This study revealed that MoHoNGT, the mobile-based web application created by our multidisciplinary team, enhanced the learning performance of NGT intubation among medical students preparing to enter their first-year clerkship. The enhancements were evident in several areas, including knowledge, self-confidence, and performance on the OSCE. While all students exhibited varying degrees of progress, those in the MoHoNGT group showed more significant improvements in both NGT intubation knowledge and the confidence scale. Participants also generally reported that MoHoNGT was user-friendly and beneficial, with high overall satisfaction. Thus, MoHoNGT effectively facilitated acquiring both knowledge and skills in NGT intubation, leaving students with a positive impression.

Novel educational technologies have narrowed the gap between theoretical learning and practical applications [29,30]. A number of innovative technology-driven training approaches have been introduced in the field of medical education. Traditionally, multimedia materials such as videos and presentations have been utilized to teach technical skills. However, prior research has shown that hands-on practice significantly improved students’ clinical reasoning abilities [29], and timely feedback is important to enhance trainees’ performance [10,13]. Therefore, video-based or presentation-based strategies, lacking both hands-on operations and real-time feedback, have been limited in effectiveness [10,31]. In recent years, augmented, virtual, or mixed reality (XR) have played significant roles in medical education. XR provides immersive and interactive scenarios facilitating decision-making and problem-solving in simulated yet safe contexts. An increasing number of studies have demonstrated that XR is a serviceable tool for training in complex clinical skills [10,32,33]. However, several issues encountered in mannikin-based learning remain unresolved with this approach, such as cost, flexibility, and limited practice duration and opportunities. XR requires additional and relatively costly equipment. Moreover, XR products utilizing outside-in tracking technology require specific spaces or locations to operate, thus reducing their accessibility [9,10]. The costs associated with developing and the programming expertise needed to develop XR software also present significant barriers to the widespread adoption of this modality [17]. On the other hand, mobile-based learning, despite being the less recent technology, is a more viable and accessible option. A smartphone integrates many input sensors and output interfaces into a handheld device while maintaining a high level of interactivity at the same time. Its untethered and portable nature allows for learning anytime and anywhere, facilitating deliberate and repeated practice, which has been shown to improve trainee performance [17,34]. Furthermore, the emergence of low-code application-building platforms enables software developers, even those with limited experience, to create practical applications through intuitive visual programming interfaces efficiently [35,36]. While mobile-based learning may be less immersive and realistic, in the current landscape of clinical skills training, it offers many practical benefits over XR, effectively and efficiently addressing various real-world challenges medical students face today.

Clinical skills training has shifted from a simple emphasis on the technical proficiency of specific procedures to a more comprehensive approach incorporating simulated scenarios. This evolution recognizes the importance of understanding the complete clinical context, strengthening critical thinking and decision-making capabilities, and enhancing patient care quality. Research has shown that simulation-based training significantly improves clinical competence and confidence among healthcare professionals [6,7,15,16]. Mobile-based learning further enriches this educational trend. Diverse clinical scenarios that vary in complexity and context can be seamlessly integrated into mobile applications, thus preparing medical students for real-world challenges, such as unexpected disruptions and complications. In addition, interactive learning experiences and immediate feedback can engage learners more effectively than static materials. For example, following NGT intubation, MoHoNGT provides several clinical scenarios in which the user must determine whether NGT placement is correct, including the X-ray image shown in Figure 2.

Several studies have investigated mobile-based education methods incorporating simulated scenarios across various domains and audiences, and our findings were generally consistent with previous results. Yamada et al. developed an automated smartphone-based suture evaluation system and successfully demonstrated its accuracy in quantifying the suturing skills of a novice [12]. Similarly, Su et al. created a mobile-based web application that yielded positive outcomes for nurse practitioners learning simple suturing techniques [17]. They also collaborated with surgeons and developed another application to facilitate education for lung tumor patients undergoing video-assisted thoracoscopic surgery [18]. In addition, Kim and Suh employed an interactive mobile application for nursing skills with systematic contents, significantly enhancing knowledge, self-efficacy, and skill performance among nursing students [19]. However, to the best of our knowledge, no existing studies have applied a mobile application featuring simulated hands-on operations and real-time feedback specifically targeting medical students in the context of NGT intubation skills training.

To develop MoHoNGT, we recorded and collected real-life images, video footage, and audio, integrating these elements into the application. We specifically designed the virtual–real fusion view, which provided a real-time side-by-side comparison of endoscopic images and the position of the NGT tip. These realistic components help bridge the gap between mobile-based learning and real-world clinical contexts, enhancing the immersion of the learning experience. Previous studies have revealed that the incorporation of real images facilitated cognitive phase learning of blind techniques, while improving memory retention, comprehension, and overall learning outcomes [24,37].

Our study had several strengths. First, compared with conventional training methods utilizing physical manikins, mobile-based learning is more flexible and cost-effective, increasing opportunities for repeated practice and reducing time gaps between training and performing real-world procedures. Furthermore, the cloud-based architecture of MoHoNGT made it platform-independent and readily accessible via any mobile device with a web browser and internet connection, all of which have become increasingly common in today’s world. In addition, thanks to a prospective randomized controlled design, all the study protocols, evaluation tools, and outcome parameters were pre-defined and well-validated, enhancing the reliability of the results. Moreover, we utilized the OSCE as an outcome parameter, effectively addressing the limitations in previous studies that relied solely on relatively subjective questionnaires for data collection [38]. Additionally, the participant population in our study was homogeneous. The medical students were drawn from a single university in the same academic year, resulting in similar levels of education, smartphone usage, and previous e-learning experience in NGT intubation. Lastly, our study is the first to employ a smartphone and a web-based interactive application to train undergraduate medical students in NGT intubation, a blind technique that has often been overlooked in the past [5].

This study came with some limitations. First, some students reported that the user interface of MoHoNGT looks cartoonish, rendering the application less professional and engaging. Indeed, our focus has been mainly on the content rather than visual appearance. Additionally, mobile-based learning cannot fully replicate the gestures involved in NGT intubation. Nevertheless, we believe that accessibility and flexibility outweigh this shortcoming. It is also worth noting that high-fidelity simulations are not necessarily superior to low-fidelity simulations in supporting learning among medical students [39]. Furthermore, MoHoNGT is compatible only with the Blink browser engine and requires a certain level of on-device processing power due to its complexity, leading to varying user experiences across smartphones and browsers. Finally, this study was conducted in one university and one academic year. Future studies with larger sample sizes covering different universities and academic years are warranted to confirm our findings. 

## 5. Conclusions

MoHoNGT was a convenient and cost-effective adjunct to the traditional training methods, significantly promoting knowledge acquisition, self-confidence, and skill performance among undergraduate medical students in NGT intubation. Overall, mobile-based learning represents a potential strategy for medical education in various fields. Future studies could further evaluate medical students’ real-world performance using the direct observation of procedural skills (DOPS), investigate the long-term retention of clinical skills with a longer follow-up period, report the results of cost-effectiveness analyses, and broaden student populations by including larger sample sizes, different universities, or varying academic years.

## Figures and Tables

**Table 1:** The demographic data of participants.

	Total (n = 73)		MoHoNGT Group (n = 32)		Control Group (n = 41)		*p*
	n	%	n	%	n	%	
Gender							0.21
Male	48	65.8	24	75	24	58.5	
Female	25	34.2	8	25	17	41.5	
Smartphone usage							0.15
<3 h/day	25	34.2	14	43.8	11	26.8	
≥3 h/day	48	65.8	18	56.3	30	73.2	
Previous e-learning experience in NGT intubation							0.28
Yes	9	12.3	2	6.3	7	17.1	
No	64	87.7	30	93.7	34	82.9	

NGT: nasogastric tube; MoHoNGT: Mobile-based Hands-on Learning System for Nasogastric Tube Intubation.

**Table 2:** Comparison of NGT intubation knowledge, the confidence scale, and the OSCE scores between the MoHoNGT group and the control group.

	MoHoNGT Group (n = 32)		Control Group (n = 41)		*p*
	Mean	SD	Mean	SD	
NGT intubation knowledge					
Pre-study	30.31	11.21	27.56	11.35	0.31
Post-study	69.06	15.32	49.02	14.80	<0.001 *
Within-group differences (t)	13.04 *		8.32 *		
Improvement ^a^	38.75	16.8	21.46	16.52	<0.001 *
The confidence scale					
Pre-study	9.44	6.14	11.49	5.86	0.15
Post-study	17.94	4.06	16.66	5.35	0.65
Within-group differences (t)	6.39 *		5.76 *		
Improvement ^a^	8.50	7.53	5.17	5.75	0.04 *
OSCE					
Post-study	98.81	5.39	91.18	14.93	0.003 *

NGT: nasogastric tube; MoHoNGT: Mobile-based Hands-on Learning System for Nasogastric Tube Intubation; OSCE: objective structured clinical examination; SD: standard deviation. ^a^ Improvement is defined as the post-study scores minus the pre-study scores. * *p* < 0.001.

## Data Availability

The original contributions presented in this study are included in the article. Further inquiries can be directed to the corresponding author(s).

## Supplementary Materials

The following supporting information can be downloaded at: https://www.mdpi.com/article/10.3390/healthcare13050546/s1, Video S1.

## Abbreviations

The following abbreviations are used in this manuscript:

NGT	nasogastric tube
MoHoNGT	Mobile-based Hands-on Learning System for Nasogastric Tube Intubation
OSCE	objective structured clinical examination
SD	standard deviation
XR	augmented, virtual, or mixed reality
DOPS	direct observation of procedural skills
